# Nuclear survivin expression is associated with HPV-independent carcinogenesis and is an indicator of poor prognosis in oropharyngeal cancer

**DOI:** 10.1038/sj.bjc.6604192

**Published:** 2008-01-22

**Authors:** S F Preuss, A Weinell, M Molitor, M Stenner, R Semrau, U Drebber, S J Weissenborn, E J M Speel, C Wittekindt, O Guntinas-Lichius, T K Hoffmann, G D Eslick, J P Klussmann

**Affiliations:** 1 Department of Otorhinolaryngology, Head and Neck Surgery, School of Medicine, University Hospital of Cologne, Cologne, Germany; 2 Department of Radiation Oncology, University Hospital of Cologne, Cologne, Germany; 3 Institute of Pathology, University Hospital of Cologne, Cologne, Germany; 4 Institute of Virology, University Hospital of Cologne, Cologne, Germany; 5 Department of Molecular Cell Biology, Research Institute Growth & Development, University of Maastricht, Maastricht, The Netherlands; 6 Department of Otorhinolaryngology, Head and Neck Surgery, University Hospital of Jena, Jena, Germany; 7 Department of Otorhinolaryngology, Head and Neck Surgery, University Hospital of Duesseldorf, Duesseldorf, Germany; 8 School of Public Health, University of Sydney, Sydney, New South Wales, Australia

**Keywords:** carcinoma, HPV, survivin, oropharynx, prognosis

## Abstract

The relationship between expression of the inhibitor of apoptosis protein survivin and the presence of high-risk human papillomavirus (HPV) in oropharyngeal squamous cell carcinoma (OSCC) remains unclear. This also accounts for its role as a predictor of survival. Therefore, we conducted a multicentre retrospective study on 106 consecutive oropharyngeal cancer patients. Human papillomavirus sequences were detected by nested PCR protocols. Survivin and p16 expression as a surrogate marker for HPV status were analysed by immunohistochemistry. Sequences of high-risk HPV were detected in 29% of cases. Prominent cytoplasmatic expression of survivin was found in 58% of cases and nuclear expression of survivin was found in 19% of the survivin-positive tumours. Nuclear expression of survivin was significantly correlated with HPV-negative tumours (*P*=0.023) and with a poor disease-free survival rate with an estimated 3-year disease-free survival probability of 35% for tumours with nuclear expression of survivin *vs* 78% for tumours with non-nuclear expression of survivin (hazard ratio=8.264; 95% confidence interval (95% CI)=2.510–27.210; *P*<0.001). In multivariate analysis, p16 expression status as well as nuclear expression of survivin were strong independent and opposing prognostic indicators of disease-free survival (hazard ratio=0.068; 95% CI=0.005–0.892; *P*=0.041 and hazard ratio=15.975; 95% CI=2.377–107.360; *P*=0.004, respectively). Our data show that nuclear accumulation of survivin correlates with HPV-independent carcinogenesis and is an independent predictor of poor survival in patients with OSCC.

Currently, there are several studies suggesting that a subset of oropharyngeal squamous cell carcinoma (OSCC) are associated with oncogenic human papillomavirus (HPV) infection, in particular HPV type 16 (Gillison and Lowy, 2004). These HPV-positive OSCC differ from HPV-negative tumours in several biological and clinical aspects including molecular alterations and prognosis (Klussmann *et al*, 2001; Smith *et al*, 2004) indicating that the former group represents a separate tumour entity (Klussmann *et al*, 2003b). Recent studies have reported that p16^INK4a^ expression is highly correlated with the presence of HPV–DNA in OSCC (Hafkamp *et al*, 2003; Klussmann *et al*, 2003a). The p16 protein is known to inactivate the function of cdk4– and cdk6–cyclin D complexes. One of the critical substrates of the G1-specific cdk–cyclin complexes is the retinoblastoma (pRb)–E2F protein complex resulting in the release of E2F upon phosphorylation (Sherr, 2001). Hence, p16 negatively regulates cell proliferation by suppression of hyperphosphorylation of pRb (Nobori *et al*, 1994). pRb also acts as a negative regulator of p16 expression (Li *et al*, 1994). Functional loss of p16 has been demonstrated in a wide variety of other tumours (Roussel, 1999). In HPV-associated carcinomas, p16 inactivation is rarely observed because the viral oncoprotein E7 inactivates the pRb protein, which is known to inhibit p16 transcription (Kim *et al*, 1998). The presence of HPV and the overexpression of p16 as a surrogate marker for HPV-positive carcinomas has been associated with a favourable prognosis in several studies (Ritchie *et al*, 2003; Reimers *et al*, 2007) and HPV-associated OSCC have also been attributed to a higher sensitivity to radiation therapy (Lindel *et al*, 2001).

Survivin, a recently characterised novel member of the inhibitor of apoptosis family, is a bifunctional protein that acts as a suppressor of apoptosis and plays a central role in cell division. Survivin has raised enormous interest in cancer research not only because it is often upregulated in malignant lesions but also because of the potential exploitation of its pathways in cancer diagnosis and therapy (Li and Ling, 2006). Its overexpression has been correlated with poor prognosis, cancer progression and drug resistance (Li and Ling, 2006). Recent reports could show a negative impact of survivin expression on survival in squamous cell carcinoma of the oral cavity (Lin *et al*, 2005; Lo Muzio *et al*, 2005). However, one study reported a conflicting result with increased overall survival rates for tumours with high survivin scores regardless of the different staining patterns such as nuclear or cytoplasmatic reactivity (Freier *et al*, 2007). There are no reports on the impact of nuclear expression of survivin on survival in OSCC, but its expression in the cell nuclei was found to correlate with a poor survival probability in oesophageal squamous cell carcinoma and small cell lung cancer (Grabowski *et al*, 2003; Lu *et al*, 2004). Moreover, recent reports found a direct relationship between survivin expression and HPV presence in squamous cell carcinomas, which suggests that HPV-associated carcinogenesis may have an effect on regulating the levels of survivin expression (Lo Muzio *et al*, 2004, 2005). It is well established that high-risk HPV E6 proteins induce the proteosome-mediated degradation of p53 and that the expression of p53 results in downregulation of survivin promoter constructs. Therefore, it is suggested that the transactivation effect of HPV16 E6 on survivin appears to be mediated by the p53 degradation pathway (Mirza *et al*, 2002; Borbely *et al*, 2006).

The aim of this study was to determine the survival impact of cytoplasmic and nuclear survivin expression in OSCC. Furthermore, we aimed to show potential interferences of the HPV-dependent carcinogenesis with cytoplasmic and nuclear survivin staining patterns.

## MATERIALS AND METHODS

### Subjects and materials

In this multicentre study, we analysed formalin-fixed, paraffin-embedded tissue from 106 consecutive patients with newly diagnosed and histologically confirmed squamous cell carcinoma of the oropharynx treated at the hospital of the University of Cologne (*n*=92) and at the University of Duesseldorf (*n*=14) between July 1998 and November 2005. Some of these cases were subjects in previously published studies (Preuss *et al*, 2007; Reimers *et al*, 2007). Patients' ages ranged from 34 to 82 years (mean age=57 years) out of which 77 patients were males (73%) and 29 were females (27%). Written informed consent was obtained from each patient and the scientific protocol was approved by the Local Ethics Committee. Tumour staging was assessed according to the 2002 American Joint Committee on Cancer staging criteria (Greene *et al*, 2002). Details of the included patients and tissues are presented in Table 1. The majority of the patients underwent a multimodal treatment approach described in detail previously (Preuss *et al*, 2007). Follow-up data were collected at periodic visits in intervals of 4–6 months at our outpatients department. Follow-up time was defined as the time from the date of the diagnosis to the date of the last visit or the date of death. The mean follow-up time was 20.3 months with a minimum of 0.33 months and a maximum of 79.8 months (median=17.2 months). Tumour specimens of all cases were obtained during surgery and the tissue was fixed in 4% buffered-formalin and embedded in paraffin by routine procedures.

All experiments were performed at the University Hospital of Cologne. Slides from all blocks were reviewed by one pathologist (UD) to select representative areas of the tumours for further processing and immunohistochemistry. The criteria for block selection were vital tumour tissue without necrosis and the presence of a front of invasion. Only blocks with estimates of at least 70% tumour cells were included.

### Sample preparation, PCR and HPV typing

Tissues were processed as described previously (Klussmann *et al*, 2001). After confirming integrity of DNA by *β*-globin gene PCR, HPV sequences were detected by highly sensitive nested PCR protocols with degenerated primers A10/A5-A6/A8 for group A (genital/mucosal) HPVs and CP62/70-CP65/69a for group B1 (cutaneous/EV) HPVs. PCR products (10 *μ*l) were separated in 2% agarose gels and were visualised by ethidium bromide staining. Human papillomavirus typing was performed as described previously (Klussmann *et al*, 2001).

### Immunohistochemical staining

A total of 101 cases in the study group were eligible for the immunohistochemical staining of p16. Formalin-fixed and paraffin-embedded biopsy samples were processed by the avidin–biotinylated–peroxidase complex method (Chem Mate Detection kit; Dako Cytomation, Carpinteria, CA, USA). Sections were deparaffinised by passage through xylene and rehydrated by a graded series of ethanol, followed by microwave treatment for antigen retrieval, which was for p16 staining two times for 7 min at 600 W in 10 mM citrate buffer (pH 6.0). After a brief rinse with Tris-buffer (BUF1), sections were incubated with the primary antibody for 25 min (p16: Ab-4, clone 16P04 Neo Markers, Fremont, CA, USA). After another brief rinse with Tris-buffer, the tissue was incubated with the biotinylated secondary antibody for another 25 min. After a brief rinse with Tris-buffer, the endogenous peroxidase was inactivated with peroxidase-blocking solution, 3 times for 2.5 min each. After rinsing, the tissues were incubated for another 25 min with streptavidin conjugated with horseradish peroxidase. Visualisation was performed three times with 3-amino-9-ethylcarbazol (AEC; Dako Cytomation) for 5 min, and then sections were counterstained with haematoxylin. Nondysplastic peritonsillar squamous cell epithelium was used as a negative control. p16 expression was classified according to a previous publication (Reimers *et al*, 2007): strong nuclear staining as well as strong cytoplasmic staining was considered positive for p16 expression and it was regarded as overexpression if it was strong and diffuse and more than 60% of the tumour cells were p16-positive.

For survivin staining, tumour sections of 6 *μ*m were cut from formalin-fixed, paraffin-embeded blocks and were mounted on glass slides with silane-treated surface and were deparaffinised. After antigen retrieval by heating in citrate buffer at 60°C in an incubator overnight these sections were treated with 3% H_2_O_2_ in methanol for 20 min to abolish endogenous peroxidase activity. Then these sections were incubated with 2 *μ*g ml^−1^ anti-survivin polyclonal rabbit (Novus Biologicals Inc., Littleton, CO, USA) at 4°C over night. Biotinylated goat anti-rabbit immunoglobulin (Dako) and streptavidin–horseradish peroxidase conjugate (Amersham Biosciences, Buckinghamshire, UK) were applied at room temperature. The sections were visualised using AEC peroxidase substrate solution and haematoxylin counterstaining. Negative control slides without primary antibody were included for each tumour section. Cytoplasmic surviving staining levels were determined according to Lo Muzio *et al* (2003): accordingly, a mean percentage of cytoplasmic-positive tumour cells was determined examining 300 cells in four areas at × 400 magnification. The tumours were assigned to one of the following categories: score 0, <5%; score 1, 5–25%; score 2, 26–50%; score 3, 51–75%; score 4>75%. Slides were considered to be nuclear positive when more than 5% of all tumour cell nuclei were stained regardless of the cytoplasmic staining levels.

Analysis of the slides was performed in a blinded fashion by two authors (UD and SP).

### Statistical analysis

Survivin, p16 and HPV status were analysed using cross-tabulations and *χ*^2^-test with the SPSS Base System, version 11.0 (SPSS, Chicago, IL, USA). Disease-free survival and overall survival rates were estimated using the Kaplan–Meier algorithm for incomplete observations. The overall survival time was defined as the interval between the date of diagnosis and the last date when the patient was known to be alive (censored) or date of death for any reason (uncensored). The disease-free survival rate was measured as the period between the date of diagnosis and the date of the last follow-up examination in which the patient was disease-free (censored), or the date of first recurrence irrespective of local, regional or distant presentation (uncensored). Univariate analysis of the various variables was performed with the log-rank test. A Cox proportional hazards ratio model was used to determine independent predictors of overall survival using factors significant on univariate analysis as covariates. A *P*-value of less than 0.05 was considered significant.

## RESULTS

### HPV status and HPV typing

Sequences of high-risk HPV were detected in DNA preparations for 30 (29%) out of 102 OSCC cases, the DNA integrity of which was sufficient. These included HPV16 in 29 (97%) cases and HPV33 in 1 case.

### Expression of p16

There were three different immunostaining patterns observed for p16 immunoreactivity, that is, strong and diffuse cytoplasmic and nuclear overexpression, weak cytoplasmic and nuclear staining and no staining for all tumour cells. In general, surrounding mesenchymal cells showed no p16 immunoreactivity, except for some weak staining found occasionally in lymphocytes and salivary glands. Nondysplastic squamous cell epithelium was always p16-negative and served as an internal-negative control. Overexpression of p16 was shown by 40% of all cases.

### Expression of cytoplasmic and nuclear survivin

**Figure 1:**
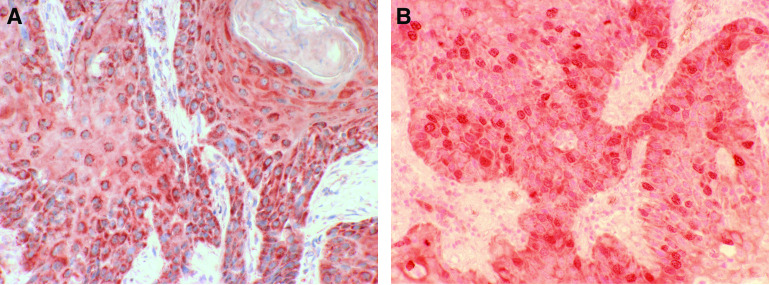
(**A**) Survivin expression in representative examples of OSCC, demonstrating strong and granular cytoplasmic staining (magnification × 400). (**B**) Distinct nuclear expression of survivin with concomitant weak cytoplasmic staining (magnification × 400).

We found a negative survivin score in 17% of all cases, score 1 in 10%, score 2 in 17%, score 3 in 25% and score 4 in 32% of all cases. In most OSCC, we found a homogeneous staining pattern throughout the entire specimen. Cytoplasmic expression of survivin (Figure 1A) was found in 83% of all cases. We found that either there was no/sporadic nuclear staining or a distinct staining intensity in more than 5% of all tumour cell nuclei regardless of the cytoplasmic-staining levels. According to the chosen cutoff point at 5%, 19% of all cases were classified as nuclear surviving-positive (Figure 1B).

There was no significant interobserver variability in the blinded analysis of the slides.

### Correlation of HPV status, p16, cytoplasmic and nuclear survivin expression

The correlation between p16 overexpression and the prevalence of oncogenic HPV–DNA in the tumour cells was highly significant (*P*<0.001). There was a significant correlation of higher cytoplasmic survivin scores in p16-negative tumours (*P*=0.0022). Nuclear expression of survivin was significantly correlated with HPV-negative tumours (*P*=0.023). There was a higher proportion of tumours with high levels of cytoplasmic survivin expression in the HPV-negative cases (*P*=0.022). There was no significant correlation of cytoplasmic survivin expression levels, positive nuclear survivin staining and p16 expression with the clinical staging variables T, N, M and tumour grading.

### Survival analysis

**Figure 2:**
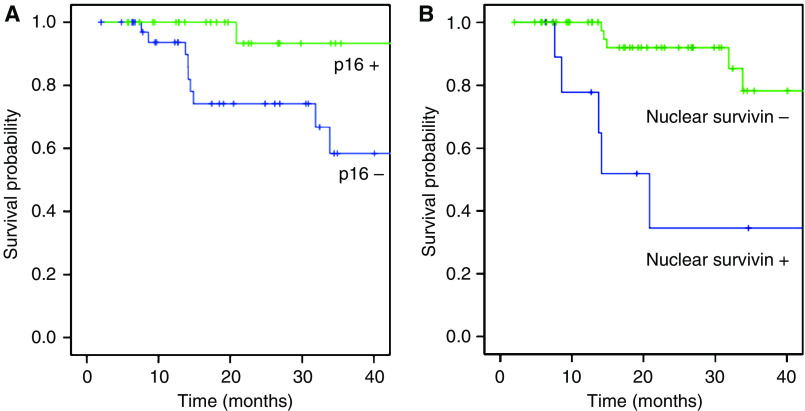
(**A**) Univariate survival analysis by p16 tumour status. Kaplan–Meier survival curves for the estimated 3-year disease-free survival probability of 93 *vs* 58%, respectively (hazard ratio=0.115; 95% CI=0.015–0.900; *P*=0.013). (**B**) Univariate survival analysis by nuclear expression for survivin. Kaplan–Meier survival curves for the estimated 3-year disease-free survival probability of 78 *vs* 35%, respectively (hazard ratio=8.264; 95% CI=2.510–27.210; *P*<0.001).

Human papillomavirus-positive tumours showed a tendency towards a better 5-year disease-free survival rate compared with the HPV-negative group (hazard ratio=0.173; 95% confidence interval (95% CI)=0.022–1.344; *P*=0.0576). The 5-year overall survival probability of HPV-positive tumours was 72%, compared with 48% for HPV-negative tumours, which was not significant (*P*=0.132). Patients with p16 overexpression in their tumours had a significantly better 3-year disease-free survival probability with 93.3 *vs* 58.4% for p16-negative cases (Figure 2A) and a better 5-year disease-free survival rate, namely, 93% compared with 44% for patients with p16-negative tumours (hazard ratio=0.115; 95% CI=0.015–0.900; *P*=0.013). Patients with p16-positive OSCC had a significantly better overall survival rate than those with p16-negative tumours with a 5-year overall survival probability of 78 *vs* 43% (hazard ratio=0.414; 95% CI=0.187–0.915; *P*=0.0244).

Higher cytoplasmic survivin expression levels were significantly associated with a poorer 5-year disease-free survival rate (hazard ratio=2.114; 95% CI=1.131–3.952; *P*=0.0387). Nuclear expression of survivin was strongly associated with an unfavourable disease-free survival rate (Figure 2B) with an estimated 3-year disease-free survival probability for OSCC exhibiting non-nuclear *vs* nuclear survivin expression of 78 *vs* 35% (hazard ratio=8.264; 95% CI=2.510–27.210; *P*<0.001). This correlation was found in HPV-negative tumours (hazard ratio=7.047; 95% CI=1.998–24.848; *P*=0.0005). In the HPV-positive tumours the same correlation was found with only one nuclear surviving-positive case in this subgroup (*P*=0.027).

### Mulitvariate analysis of prognostic factors

Using the Cox proportional hazards model, we performed a multivariate analysis to assess the independent predictive value of all significant markers in overall- and disease-free survival, that is, nuclear survivin staining and p16 expression and TNM stage. p16 expression as well as nuclear expression of survivin were independent and significant prognostic factors for disease-free survival in this model (hazard ratio=0.068; 95% CI=0.005–0.892; *P*=0.041 and hazard ratio=15.975; 95% CI=2.377–107.360; *P*=0.004, respectively). The overall survival probability was significantly affected by the factors p16 expression status and M stage (hazard ratio=0.309; 95% CI=0.113–0.848; *P*=0.023 and hazard ratio=8.040; 95% CI=2.763–23.391; *P*=0.0001, respectively).

## DISCUSSION

Previous studies have shown that at least one-third of OSCC are infected by oncogenic HPV, predominantly HPV type 16 and a large meta-analysis showed a proportion of HPV-positive OSCC of 36.6% (Kreimer *et al*, 2005). We found HPV-positive OSCC in 29% of our patients which is in line with a previous publication of our group (Reimers *et al*, 2007). The slightly lower rate as compared to other studies might be the result of different socio-economic profiles and risk factors of the patients. Several studies showed a distinct biological behaviour of the HPV-positive subset of oropharyngeal tumours, resulting in a more favourable prognosis (Li *et al*, 2003). Recently, the specific T-cell response to HPV16 E7 epitopes in subjects with HPV16 E7 expression and p16-positive OSCC was shown (Hoffmann *et al*, 2006). p16 upregulation is also present in HPV-related uterine cervical lesions, and it is likely that p16 is upregulated in HPV-positive tumours due to the interaction of the HPV16 E7 oncogene product with the pRb protein. This suggests that p16 overexpression is most likely the result of transcriptionally active HPV infection and in previous studies, p16 expression was highly correlated with the HPV status in OSCC (Klussmann *et al*, 2003a). In line with this, we found that p16 overexpression was highly correlated with the presence of HPV–DNA in this series of OSCC (*P*<0.0001). p16 overexpression was an independent indicator of a favourable disease-free survival, which is in line with other reports (Mellin *et al*, 2000).

Targeting survivin may provide a novel perspective in cancer therapy by simultaneously disabling multiple signalling circuitries. Currently, several clinical trials targeting survivin with various approaches ranging from immunotherapy to antagonists are under way and might be broadly applicable to different tumours (Altieri, 2006). High levels of cytoplasmic survivin expression have been reported previously in OSCC (Weinman *et al*, 2003) with similar staining patterns as shown in our study in which 58% of all tumours prominently expressed survivin in the cytoplasm. Here, we found a significantly inverse correlation between cytoplasmic survivin expression and HPV-associated carcinomas. Human papillomavirus-positive tumours and p16 overexpression were significantly associated with lower scores for cytoplasmic survivin expression (*P*=0.028 and *P*=0.022, respectively). Cell culture experiments suggest that expression of high-risk HPV E6 proteins lead to survivin overexpression by the proteosome-mediated degradation of p53, which inhibits p53-mediated downregulation of survivin promotor constructs (Munger and Howley, 2002). However, inactivation of p53 has not only been found in HPV-associated carcinoma but also in almost all OSCC. In fact, in the carcinogenesis driven by environmental toxins such as tobacco and alcohol, p53 mutations are more frequently found with subsequent deletion of p53 (Balz *et al*, 2003). Moreover, biallelic loss of the gene or transcriptional silencing of p53, both of which have been reported in OSCC cell lines, results in a complete loss of transcript in tumour cells (Hauser *et al*, 2002). These findings might explain the positive correlation of cytoplasmic survivin overexpression and HPV-negative tumours in our series.

Survivin overexpression has been shown to be related to a poor survival probability in squamous cell carcinomas of the oral cavity (Lo Muzio *et al*, 2005). However, in a recent paper the contrary finding that high survivin expression levels predict a favourable overall survival in oral squamous cell carcinoma was reported. This was most distinctly found in patients who were treated with radiotherapy. Therefore, the authors concluded that survivin expression might be used as a marker to predict the response to radiotherapy (Freier *et al*, 2007). In our series, cytoplasmic survivin overexpression was significantly correlated with poor disease-free survival rates in univariate analyses. However, the subgroup of patients that received primary radiotherapy was too small for statistical evaluation of differences in survival probabilities. In line with several studies, we found that in OSCC, survivin exists in two subcellular pools, that is, cytoplasmatic and nuclear. All tumours with immunoreactivity for survivin showed cytoplasmic expression and 19% showed nuclear expression of survivin. As survivin mediates the regulation of both cell viability and cell division, the nuclear pool of survivin is likely to be involved in promoting cell proliferation, whereas the cytoplasmic pool of survivin may participate in controlling cell survival (Li, 2003). However, the molecular mechanisms underlying nuclear survivin expression in tumours are not entirely understood. Active nuclear import is mediated by nuclear localisation signals (NLS), which interact with import receptors (Weis, 2003). Mutations in the NLS or enhanced binding to nuclear components may account for pronounced nuclear survivin (Engels *et al*, 2007). The nuclear expression patterns of survivin were significantly correlated with HPV-negative tumours in our series, which suggests that the HPV-dependent carcinogenesis interferes with the active nuclear import of survivin. Several studies found that nuclear expression of survivin predicts poor survival probabilities in human cancer (Grabowski *et al*, 2003; Lu *et al*, 2004) but this finding remains controversial as papers on identical tumour entities show correlation of nuclear survivin expression with a favourable prognosis (Vischioni *et al*, 2004). So far, no study investigated this correlation in OSCC. The nuclear expression of survivin in our study strongly correlated with poor disease-free survival probabilities in univariate as well as multivariate analyses (*P*<0.001 and *P*=0.004, respectively). However, considering the short follow-up time with a median of 17.2 months and the relatively small number of patients in the nuclear surviving-positive group, the significance of these results is somewhat limited.

In conclusion, our findings show an expression of survivin in the majority of OSCC. The cytoplasmic and particularly the nuclear survivin expression predict a poor survival outcome in these patients. The survivin expression patterns seem to be influenced by the different molecular pathways of carcinogenesis and nuclear survivin expression seems to be increased in HPV-negative tumours.

## Figures and Tables

**Table 1:** Clinicopathological characteristics of the patients

**Characteristics**	**No. of patients**	**%**	***N* (total)**
*Sex*			106
Male	77	73	
Female	29	27	
			
*Age*			
Median	57		
Range	35–83		
			
*T-stage*			106
1	25	24	
2	31	29	
3	17	16	
4	31	29	
			
*N-stage*			105
0	18	17	
1	21	20	
2a	3	3	
2b	30	28	
2c	16	15	
3	14	13	
			
*M-stage*			103
0	95	92	
1	8	8	
			
*Treatment*			100
Surgery	76	76	
Surgery+RT/RCT	55	55	
RT/RCT alone	24	24	
			
*Survivin score*			94
0	16	17	
1	9	10	
2	16	17	
3	23	25	
4	30	32	
			
Survivin nuclear expression			94
+	18	19	
−	76	81	
			
*p16 overexpression*			101
+	40	40	
−	61	60	
			
*HPV detection*			102
+	30	29	
−	72	71	

RCT=radiochemotherapy; RT=radiotherapy.
